# Critical lure source details are “correctly” attributed to both directly related and mediated lists

**DOI:** 10.3389/fpsyg.2025.1529070

**Published:** 2025-02-07

**Authors:** Alexa E. Tringali, Mark J. Huff

**Affiliations:** School of Psychology, University of Southern Mississippi, Hattiesburg, MS, United States

**Keywords:** Deese–Roediger–McDermott paradigm, activation-monitoring theory, fuzzy-trace theory, global-matching model, source-monitoring, false memory

## Abstract

Studying lists of associatively related words often produces false recognition of non-studied critical lures (CL). This false memory illusion can be found both when word lists are directly related to a CL as in the DRM paradigm (e.g., water, bridge, run, for the CL *river*), and when words are indirectly related to CLs via non-presented mediators (e.g., faucet[water], London[bridge], jog[run], for the CL *river*). Mediated false memory is strong evidence for activation-monitoring processes over gist extraction as mediated lists lack a consistent gist theme. In the present study, we evaluated whether context details (font color) of studied lists are attributed to CLs when they are falsely recognized. Participants studied directly related and mediated word lists presented in one of two font colors, followed by a source test which required specification of the font color for recognized test items. When CLs were falsely recognized, participants were able to correctly identify the font color of the CL’s origin list for both list types at a higher rate than incorrect identification. Because mediated false recognition reflects implicit activation, this pattern indicates activation processes may include both semantic and perceptual source details.

## Introduction

Despite the capacity to encode, store, and retrieve accurate representations from the past, human memory is highly imperfect, leading to memory errors (Nairne, 2010; Schacter, 2002). While some errors are benign, such as misremembering the name of an acquaintance or the date an event occurred, others are more serious, such as misremembering the details of an eyewitnessed crime. Memory errors are generally classified into two types: omission errors and commission errors. Omission errors refer to misremembering due to forgetting, and commission errors refer to remembering details that did not occur or events differently than their original occurrence (McDaniel et al., 2009). A common method to produce and evaluate commission errors is through Deese/Roediger-McDermott (DRM) false memory paradigm (Deese, 1959; Roediger and McDermott, 1995). In this paradigm, lists of associates (i.e., bed, pillow, rest) are all linked to a non-presented critical lure (CL; i.e., *sleep*). At test, false recall of CLs can exceed 50% and false recognition can approximate 80% (Huff and Bodner, 2019; Roediger and McDermott, 1995). This robust false memory rate has been dubbed the *DRM illusion* (see Gallo, 2010, for review).

Several theories have been proposed to explain the DRM illusion, but the three most prominent ones are fuzzy-trace theory (FTT), the global-matching model (GMM), and activation-monitoring theory (AMT). FTT argues participants generate two separate memory traces at study—a verbatim trace and a gist trace (Brainerd et al., 1995). The verbatim memory trace includes the exact details of a memory (i.e., the exact words on the list) whereas the gist trace only contains the overall theme of a memory set (i.e., the general theme of the list). After delay, verbatim memory declines quickly while the gist trace persists. Because DRM CLs lack a verbatim trace as they were not studied, the DRM illusion occurs due to the availability of the gist trace. Consistent with the time course of verbatim and gist traces, studies have shown the DRM illusion can persist for weeks to months with little change despite robust declines in memory for correctly studied words (Reyna and Brainerd, 1998; Reyna et al., 2016; Seamon et al., 2002). Gist information, therefore, appears to be stable over retention intervals and is less susceptible to forgetting processes than verbatim information.

Separately, the GMM posits multiple memory traces and contextual information contribute to the production of false memories (Arndt, 2010, 2015; Arndt and Hirshman, 1998). In this framework, a memory trace is stored for each item studied. During retrieval, each memory candidate is compared to all memory traces stored at encoding, and an activation value is created based on similarities between the memory candidate and the stored trace(s). These similarities can be superficial, like phonological or orthographic features, or deeper, such as semantic meaning (see Coane et al., 2021, for review). Activation values are then summed to create a total value that represents the memory activation of the test item. When the memory activation meets a threshold, the memory candidate is reported as studied. In the DRM paradigm, exposure to the CL at test can create an activation value sufficient to trigger a false retrieval. Furthermore, GMM argues that when a memory candidate approximates both item and context details of a stored memory trace, it becomes activated via the interaction of multiple cues influencing the retrieval process (i.e., interactive cueing; Clark and Gronlund, 1996). This argument suggests context details are encoded, and lure items can cue the encoded context details of its associates. If the source detail of a memory candidate matches the encoded source detail of the associates, a correct source attribution is more likely to occur.

Finally, AMT proposes a two-stage process to account for false memories which includes an activation phase at encoding, and a monitoring phase at retrieval (Roediger et al., 2001). Items stored in a semantic network become activated at encoding, and this activation implicitly spreads to neighboring nodes via an automatic spreading-activation process (Collins and Loftus, 1975; Howe et al., 2009; Underwood, 1965). The more strongly associated items are tightly clustered within the network and likely to be activated via spreading activation. Because the CL is strongly related to the studied list items, which are activated during their presentation, spreading activation processes also activate the CL. In the second stage of AMT, during retrieval, memory decisions are based on the amount of source or contextual information available for a memory candidate via source monitoring, which can determine if a memory is veridical or internally generated, as in the case of the CL. If the source-monitoring process fails, a test item may not be easily dismissed as never having been seen previously. The DRM false memory illusion is, therefore, a product of both implicit spreading activation of the CL at encoding and the inability to effectively monitor for the source of this activation at test.

Backward Associative Strength (BAS) is a common metric for assessing the role of implicit spreading activation in the DRM illusion and is computed as the associative strength between the critical lure and the list items. It can be calculated using the Nelson et al. (2004) word association norms. Using a multiple regression analysis, Roediger et al. (2001) demonstrated that BAS was the strongest predictor of false recall and the second strongest predictor of false recognition (the strongest predictor being correct recognition). The theoretical basis behind this finding is that BAS serves as an index of spreading activation, which is strongly related to both CL false recall and false recognition.

### List-type effects in the DRM paradigm

Even though studies have shown support for all three accounts of the DRM illusion, each one emphasizes the role of semantic relatedness in the creation of these errors. To better tease apart these theories, researchers have developed different types of studied word lists consisting of different properties designed to test different processes. One such studied list type is a homograph list in which list items are related to different meanings of a single CL. For example, a list for the CL *right* would contain words like *wrong*, *handed*, *starboard*, *correct*, and *left*. Some of the words are related to one meaning of the word (i.e., direction), while others are related to another meaning (i.e., correctness). When homograph list words are blocked together by meaning, a clear and coherent gist is more distinguishable. However, alternating the order of meanings within the studied list can make gist extraction difficult as the different themes would not be displayed continuously. Homograph lists, therefore, provide a test of gist extraction in FTT as the list organization can affect the strength of available list themes.

To evaluate whether gist disruption affected false memory rates, Hutchison and Balota (2005) compared homograph lists in which the studied list items were either blocked or alternated (i.e., interleaved) by meaning. Both variants featured words that converged on a CL, but alternated lists, compared to blocked lists, are less thematically continuous, reducing the gist strength. In their study, which used immediate memory tests, homograph list variants produced equivalent false memory rates when presented in blocked or alternated presentations that were matched on BAS. However, in a subsequent study, false memory for homograph CLs from blocked lists were greater (Huff et al., 2015) when tests were delayed over a retention interval that encouraged the use of gist-based information at test (cf. Reyna et al., 2016). False memory homograph lists appear to support both an activation-based and gist-based process, for immediate and delayed retention intervals, respectively.

Another list type developed to compare potential false memory mechanisms is the mediated list. Mediated lists consist of words indirectly associated to each other and the CL. For example, the CL *river* includes DRM list items such as: *water*, *bridge*, and *run*. On mediated lists, however, words are only indirectly related to the CL through DRM list items which serve as mediators. For example, *faucet* is related to *water*, and *London* is related to *bridge*, but neither faucet nor London are directly related to each other nor to the CL *river*. Importantly, mediated lists eliminate the thematic consistency necessary to develop a gist trace as the list words are unrelated to each other and only indirectly related to the CL. In an initial study, Huff and Hutchison (2011) explored how meditated lists affected implicit spreading processes in three experiments. On a recognition test, mediated word lists produced false alarms for the CL at a rate higher than unrelated test distractors, even controlling for lexical and semantic characteristics of the distractors. These findings suggest an automatic spreading activation process took place at encoding of the word lists leading to false recognition of mediated lures.

Subsequently, Huff et al. (2012) further evaluated mediated false memory effects by comparing false recognition rates of directly related DRM lists and mediated lists when participants completed a series of interpolated tasks immediately after study. Specifically, following study of either a directly related DRM list or a mediated list, participants completed either an arithmetic task, a free recall test, a free recall test with a warning not to recall a CL, or guessing task in which participants were asked guess the CL of the list while not explicitly retrieving any of the items from the study list. On a final recognition test, DRM CL false recognition rates were highest for participants given the free recall test only and lower for participants given the free recall test with a CL warning or the guessing task. This pattern indicated that the direct association between list items and the CL were strong enough to be detected and mitigated under instruction to monitor and/or identify the CL. Conversely, rates of false recognition for mediated CLs increased when participants were given a warning or asked to guess the CL relative to the recall test. This result was termed the *ironic effect of guessing*, because identification of the CL should have aided participants at monitoring for CLs at test, but this finding only occurred when CLs were identifiable on directly related DRM lists. The researchers argued that mediated false recognition can be enhanced following interpolated tasks that strengthen semantic associations between list items and the corresponding CL. Completion of a recall test and attempts to guess a CL from a mediated facilitated CL activation via spreading activation, which was difficult to monitor on a subsequent recognition test.

Additional evidence for activation processes in mediated lists has been found using a semantic-priming paradigm (Huff et al., 2021). When participants completed either a speeded naming or semantic classification task immediately after studying a mediated list, responses to the CL were facilitated when the CL was presented both early in a test list and late in a test list, though priming for the CL was greatest in the first test position and decayed in later positions, though priming was not eliminated. The pattern in which CL activation is strongest initially but decays over an increasing timespan is consistent with activation-based processes.

The lack of a consistent gist theme in mediated lists precludes FTT as a viable explanation for list-based false memory effects. Additionally, for GMM to account for the pattern, it must be assumed that the features of mediated list items sufficiently overlap with the CL to produce false recognition (Coane et al., 2021; Hintzman, 1986). For example, Coane et al. (2021) argued the mediated item *faucet* and the CL *river* share the feature of water thus *faucet* could be falsely recognized due to its similarity with *river*. Similar to AMT, this argument implies that mediated lists would elicit rates of higher correct false memory attribution than incorrect false memory attribution rates, but these rates would be lower than direct lists because the feature overlap is weaker than with DRM lists even though mediated items share feature overlap with the CLs. However, GMM struggles to account for the ironic effect of guessing reported by Huff et al. (2012) in which false alarms for CLs were greater for interpolated guessing and warning tasks. The interpolated guessing task increased false alarms for mediated list CLs due to such tasks strengthening the semantic association between the CL and mediated list items. These tasks do not, however, strengthen feature overlap, thus GMM would argue that false alarm rates should stay the same across tasks (e.g., arithmetic vs. guessing).

### Source-monitoring and false memory

Whereas mediated lists have been used to investigate the development of false memory illusions using lexical materials, other studies have evaluated source monitoring processes that may contribute to these errors. Source-monitoring is a process that aids in the ability to identify a true detail or memory and dismiss false or internally generated ones. Source-monitoring judgments involve recollection of contextual details of a potential memory candidate and assessing whether these recollected details are consistent with details expected of a true memory (Ball et al., 2014; Johnson et al., 1993).

Smith et al. (2022) examined the effect of distinctive encoding strategies on source-monitoring and mitigating false memories using mediated and homograph lists. Distinctive encoding generates cues that aid monitoring at retrieval (Schacter and Wiseman, 2006). Researchers gave participants item-specific, relational encoding, or read-only instructions for studying a series of homograph and mediated lists before taking a recognition test. Smith et al.’s results demonstrated that item-specific encoding can increase veridical recognition and decrease false recognition of critical lures by enhancing monitoring based on a signal-detection estimate of monitoring processes. This pattern aligns with previous literature on the distinctiveness heuristic following item-specific encoding (e.g., Einstein and Hunt, 1980; Glanzer and Adams, 1990; Huff and Bodner, 2013; see Huff et al., 2015, for review). Overall, the results do not support FTT, and instead, indicate that item-specific reductions in false memories can occur for lists that either disrupt or lack a gist trace.

Although the number of correct source details recollected increases the likelihood individuals can discriminate between a truly experienced event and, in associative false memory studies, an internally generated one, the ability to disqualify a CL as having not been studied becomes difficult if source details for a studied item can be bound to a CL. To that end, Hicks and Hancock (2002) examined how the associative strength between list items and critical items affected false recall and source attributions. The researchers auditorily presented participants with a series of DRM lists read either in a male or female voice. In the within-list experimental condition, half of each list contained items with high average BAS presented exclusively in one voice and the other half of each list had items with a lower average BAS presented in a different voice.

List items with strong BAS in the Hicks and Hancock (2002) study yielded high recall rates (consistent with Roediger et al., 2001), and the CLs were likely to be attributed to the speaker source used to present the strong BAS list half. Conversely, the list half with a weaker BAS displayed no such trend. The researchers suggested that the high probability of the CL being activated at encoding for high BAS list items enables the source details associated with the list item to link to the CL’s activation. When the CL is activated at encoding, it is also associated with the same source characteristics as the studied list items, resulting in a source attribution of the CL’s origin list. A similar pattern was reported by Bodner et al. (2017) in which participants studied DRM lists that were encoded using one of three different encoding tasks including anagram generation, mental imagery, or read-only intentional encoding instructions. Participants attributed the origin encoding task to falsely recognized lures and this pattern was stronger for more deeply encoded anagram generation and mental imagery tasks than the read only task. Collectively, source details of a CL’s origin list are attributed to lists that are both high in BAS and deeply encoded.

To explain how GMM could account for the DRM illusion, Arndt (2015) examined how Forward Associative Strength (FAS)—the associative strength between a list item and critical lure—and BAS influenced false memories. Participants were instructed to study weak BAS/FAS associates in one font and strong BAS/FAS associates in a different font. The results indicated that CLs were more often attributed to the source of the strong BAS items, consistent with Hicks and Hancock (2002). Arndt argued that these findings are also consistent with GMM because it suggests BAS and FAS, which index memory trace similarity (though they also index associative activation), are equally likely to predict false recollection. However, GMM itself provides no specific mechanism in how source details become bound to CLs in the DRM paradigm, and it is equally plausible that spreading activation processes can allow for source details to become associated and bound to CLs during study. Given that mediated lists provide strong evidence of activation-based processes, evaluating whether source details can be bound to indirectly related CLs could be informative regarding the role of spreading activation in binding source details at study.

### Current study

The goal of the present study was to investigate whether source details for the origin list of directly related and mediated lists could be attributed to CLs on a source-recognition test. Because false memories for mediated lists lack a gist trace and have minimized feature overlap, source detail attributions made for these lists should follow the same pattern as direct lists if spreading activation influences source-monitoring processes and contributes to false memory patterns. By comparing recognition accuracy and source detail attributions for direct and mediated lists, this study was designed to determine how source details are processed for CLs and explore the mechanisms that contribute to associative false memory illusions.

We first expected that direct and mediated lists would produce a reliable false memory illusion seen in the literature (e.g., Coane et al., 2021, for review), which is inconsistent with FTT, but consistent with the spreading activation process in AMT. If a spreading activation is occurring, mediated lists would be expected to elicit the same pattern of accurate source attributions/indications for the CL compared to the direct lists (Huff et al., 2011; 2021). Due to the indirect associations to CLs in mediated lists, however, we expected to see this pattern as a reduced rate compared to DRM lists. If accurate source attributions for the CL’s origin list occur for direct lists but not mediated lists, this pattern would align more with feature overlap (via GMM) versus spreading activation (via AMT). We predicted that participants would correctly report source details for the CL’s origin list at a rate greater than they would incorrectly report source details for the CL’s origin list for directly related DRM lists and mediated lists, which would support a spreading activation process.

## Method

### Participants

Ninety-one undergraduates were recruited via The University of Southern Mississippi’s participant pool. The age of participants ranged from 18 to 39 years old (*M* = 20.14, *SD* = 3.00). Sixty-nine participants identified as female, 21 as male, and 1 as other. Participants were compensated with partial course credit for their participation. All participants were fluent in English with normal or corrected-to-normal color vision. A sensitivity analysis conducted using G*POWER (Faul et al., 2007) revealed that our sample had adequate power (0.80) to detect effect sizes of *d* = 0.30 and larger for all comparisons reported.

### Materials

Twenty-four DRM lists and 24 mediated lists were taken from Huff et al. (2012) and served as study materials. Each list consisted of 15 items. The direct lists were DRM lists in which all study items were directly related to a CL (e.g., cat, night, bottom, for *black*). The mediated lists were indirectly related words that converged upon a CL (e.g., meow, day, basement, for *black*), through non-presented mediators (the DRM list words). The 48 lists were subdivided into four versions of 12 lists, with six DRM and six mediated lists. These versions were counterbalanced across participants. In each version, six lists (three direct and three mediated) were presented in blue font, whereas the other six lists were presented in red font. Color was also counterbalanced, such that each list type was presented in red or blue font across participants.

A source recognition test was created, which included 96 total items. These test items were subdivided into 36 studied list items (18 direct and 18 mediated taken from positions one, eight, and ten in each list), 12 CLs (six direct and six mediated), 36 unstudied control items (taken from another counterbalanced set in the same list positions) and 12 control lures (six direct and six mediated from another counterbalanced set). Test items were presented in a newly randomized order for each participant. All test items were presented in a black-colored font.

### Procedure

Following informed consent, participants were told they would be shown a series of 12-word lists, which would be followed by a memory test. Participants were instructed to pay attention to the color of each word as well as the word itself. Using SuperLab 6.0 software (Cedrus, 2024), words were presented individually on a computer screen with each word appearing for 2000 ms with a 500 ms interstimulus interval. After each word list was presented, a blank screen with the words “Next List” appeared for 2000 ms. Direct and mediated lists were presented in the same order for each participant, though the studied items in each list were presented in a newly randomized order.

Immediately following study, participants completed a source-monitoring recognition test. Participants were presented with test items individually on the computer screen and instructed to classify each item as presented in blue or red font if the word was studied, or to classify the word as “new” if the word was not studied. Participants were provided with a 3-button response box with either blue or red color options, or a button labeled “N” for new. The test was untimed, and participants were instructed to respond quickly but without compromising accuracy.

## Results

Paired sample *t*-tests compared mediated and direct lists for overall correct and false recognition as well as correct and incorrect source attributions for list items and critical lures. Table 1 reports the mean correct and false recognition rates as a function of list type. Overall correct recognition was higher for direct lists than mediated lists (0.77 vs. 0.72 for mean correct recognition for direct and mediated lists, respectively), *t*(90) = 4.04, Standard Error of the Mean (*SEM*) = 0.02, *p* < 0.001, *d* = 0.42. Overall false recognition for CLs was also higher for direct lists than mediated lists (0.75 vs. 0.53), *t*(90) = 9.26, *SEM* = 0.02, *p* < 0.001, *d* = 0.97.

**Table 1:** Mean (±95% CI) proportions of “old” recognition responses to studied list items and critical items based on list type (direct or mediated).

List and item type	Direct list	Mediated list
	*M* (+/- 95% CI)	*M* (+/- 95% CI)
Overall recognition		
List item	0.77 (0.04)	0.72 (0.04)
Critical lure	0.75 (0.05)	0.53 (0.06)
Controls	0.44 (0.05)	
Correct source		
List item	0.48 (0.04)	0.41 (0.03)
Critical lure	0.48 (0.05)	0.30 (0.05)
Incorrect source		
List item	0.29 (0.03)	0.31 (0.03)
Critical lure	0.27 (0.04)	0.23 (0.04)

*N = 91.*

In terms of source accuracy for studied list items, on direct lists, participants attributed the color source on directly related lists significantly more often than the incorrect color source (0.48 vs. 0.29), *t*(90) = 6.50, *SEM* = 0.03, *p* < 0.001, *d* = 0.68. Similarly, on mediated lists, participants were more likely to attribute the correct color source than attribute the incorrect color source (0.41 vs. 0.31), *t*(90) = 4.08, *SEM* = 0.02, *p* < 0.001, *d* = 0.43. Critically, participants were also likely to accurately attribute the font color source from the studied origin list to the corresponding CL than inaccurately. This pattern occurred for both direct lists (0.48 vs. 0.27), *t*(90) = 5.46, *SEM* = 0.04, *p* < 0.001, *d* = 0.97, and for mediated lists (0.30 vs. 0.23), *t*(90) = 2.10, *SEM* = 0.03, *p* = 0.04, *d* = 0.32.

## General discussion

The goal of our study was to evaluate whether intrinsic contextual details presented with DRM and mediated study lists could be attributed to non-studied CLs. Following study of directly related DRM lists and indirectly related mediated lists presented in one of two font colors, participants completed an immediate source-recognition test, which required the specification of the color for all items recognized as studied. Participants were able to correctly recognize the source color for correctly studied list items for both direct and mediated lists, and importantly, they correctly attributed the color source from the origin list at a higher rate than incorrectly for CLs from direct and mediated lists. Although attributions of source details have been reported for CLs from DRM lists previously (e.g., Hicks and Hancock, 2002), this pattern has not been shown for mediated lists, which do not share a direct association to a CL.

We used direct and mediated lists to evaluate theoretical processes regarding how intrinsic contextual details may become bound to CLs. Because fuzzy-trace, global-matching, and activation-monitoring theories all adequately account for the DRM false memory illusion, the finding that participants can attribute contextual details from the origin list in direct lists indicates that any one of these mechanisms might account for the binding of context to CLs. For mediated lists, however, the list items are only indirectly related to CLs, lacking a consistent list theme necessary for gist extraction and feature overlap between the stored memory trace and the test item necessary for a global-matching process. Instead, mediated false memory is likely the result of implicit spreading activation. Our finding that source details from a mediated lure’s origin list are more likely to be correctly than incorrectly attributed/indicated suggests that implicit associative processes can involve the binding of source details to non-studied lures.

Although our data suggest that context details can be bound to CLs when only associative processes are in operation, it is important to note that our results do not eliminate gist and feature matching processes for binding source details when these characteristics are available within a study list. For instance, when a participant falsely recognized a CL, we found that participants are more likely to identify the source of lure’s origin list based for directly related lists (62.60% attribution rate for falsely recognized lures) than mediated lists (52.50%), *t*(87) = 2.09, *SEM* = 0.03, *p* = 0.04, *d* = 0.22. This pattern suggests that thematic or matching processes available via direct lists drive processing that could operate in addition to implicit association to bind contextual details from the origin list to the CL and strengthen the availability of source details. Future research should explain whether high attribution rates for direct lists are due to association and gist extraction, association and trace matching, or a combination of all three factors.

The consideration of feature overlap is further complicated by the interplay between conceptual similarity and associative strength. For instance, while the weaker BAS of mediated lists produced a decreased rate of false recognition versus direct lists as expected, false recognition and source attributions were still made for mediated and direct test. Arndt’s (2015) argument for GMM provides a potential explanation, proposing that activation is not the only attribute influencing false memory, as conceptual similarity also facilitates memory traces. By this logic, variables that increase the similarity between a memory trace and memory candidate also increase false recognition. This argument may account for the rates of appropriate source attributions for falsely recognized CLs being higher for direct than mediated lists in the current study because direct lists have higher conceptual similarity. However, there is no experimental way to fully separate conceptual similarity and associative strength as the two are naturally confounded. Therefore, neither associative strength nor conceptual similarity can be ruled out as an explanation for false memories of source details for CLs at higher-than-chance rates for mediated lists.

There are, however, experimental ways in which we can explore the role source monitoring plays in false recollection by manipulating how contextual details (e.g., extrinsic details like study environment) influence the ability of studied items to be bound to CLs. To elaborate, font color is an intrinsic detail and having participants choose between two color options facilitates guessing the correct font color. Future studies may want to expand on the amount or quality of source information available at encoding. For example, adding additional font colors during the encoding phase could make source detail recollections more difficult (e.g., red, blue, green, purple). By manipulating the difficulty of retrieving extrinsic and intrinsic source information, future research can more precisely delineate how effectively people distinguish true from false source memories.

Another procedural detail that could be explored in future research is when the source detail (i.e., color) is presented. In the current study, every list was presented either in red or blue with no interleaving within each list. This procedural detail could have facilitated the binding of source details to the CL’s origin list for mediated and direct lists alike because the color detail was bound to the whole list rather than each individual item. List-wise coloring may also explain why there were higher accurate source attribution rates for direct lists than mediated lists given that the thematic/feature overlap of DRM lists are more distinct making it easier to associate the list with the color. Such reasoning falls in line with Hicks and Hancock’s (2002) argument that source judgments could potentially be made based on the participant’s estimate of when they saw each list (temporal position) compared to individual item details. Therefore, future studies could present various color fonts within each list as well as between to determine whether it affects source memory accuracy.

Although our study was successful at producing elevated rates of both DRM and mediated false memory, we note that these patterns were found only under immediate test conditions. The tendency of source details from the study lists to be bound to the CLs should be more likely seen on an immediate test than on a delayed test, especially for mediated lists. We adopted this procedure as it has been shown that longer intervals between study and test can reduce source recollections, which is similar to the forgetting that occurs for item recognition (Bornstein and LeCompte, 1995). Binding source details from the study list to the CL would be more likely seen on an immediate test than a delayed test, especially for mediated lists, however, this has not yet been examined. Of course, an important question is whether source information remains available for CLs after a delay and if this information is available similarly for both direct and mediated lists. If source details become bound to CLs via a spreading activation process, one possibility is that source details may also decay over time consistent with spreading activation. Thus, in the absence of a gist trace or feature overlap, which can aid item recognition over time (Reyna et al., 2016; Seamon et al., 2002), correct source attributions for CLs may decline more robustly for mediated than direct lists after a delay. Future research should test longer retention intervals to further our theoretical understanding of how source details of CLs become bound and are accessed at different time points.

## Conclusion

The current study compared source recognition accuracy for mediated and direct DRM lists displayed in either a red or blue colored font. We chose to compare mediated and direct lists because false memory illusions for mediated lists are strong evidence of activation-monitoring processes. Evaluating whether source details can be bound to indirectly related CLs lends insight into which theory best accounts for the DRM illusion. We found false recognition for CLs from DRM and mediated lists, lending support for an activation-based process. Additionally, results demonstrated that participants were able to accurately attribute contextual details from a CL’s origin list for both list types. These findings are important because they provide evidence that an associative activation process not only results in a semantic activation of the CL but also binds contextual details from the origin list to the CL that participants can access at test. The finding that accurate list attributions can occur on mediated CLs indicates that this binding process can occur when the list lacks a consistent theme (i.e., gist) and when the list lacks an overlap of features (i.e., global matching). Determining whether associative activation is the only mechanism for binding contextual details to CLs or whether the presence of a coherent theme or feature overlap can aid this process is an important question for future research to answer.

## Data Availability

The datasets presented in this study can be found in online repositories. The names of the repository/repositories and accession number(s) can be found below: https://osf.io/p4dsk/.
